# DNPEP is not the only peptidase that produces SPAK fragments in kidney

**DOI:** 10.14814/phy2.13479

**Published:** 2017-11-09

**Authors:** Rainelli Koumangoye, Eric Delpire

**Affiliations:** ^1^ Department of Anesthesiology Vanderbilt University School of Medicine Nashville Tennessee

**Keywords:** Antibody specificity, CRISPR/cas9, DNPEP knockout, mouse model validation, proteolytic cleavage, Ste20p‐like kinases

## Abstract

SPAK (STE20/SPS1‐related proline/alanine‐rich kinase) regulates Na^+^ and Cl^−^ reabsorption in the distal convoluted tubule, and possibly in the thick ascending limb of Henle. This kinase phosphorylates and activates the apical Na‐Cl cotransporter in the DCT. Western blot analysis reveals that SPAK in kidney exists as a full‐length protein as well as shorter fragments that might affect NKCC2 function in the TAL. Recently, we showed that kidney lysates exerts proteolytic activity towards SPAK, resulting in the formation of multiple SPAK fragments with possible inhibitory effects on the kinase. The proteolytic activity is mediated by a Zn^2+^ metalloprotease inhibited by 1,10‐phenanthroline, DTT, and EDTA. Size exclusion chromatography demonstrated that the protease was a high‐molecular‐weight protein. Protein identification by mass‐spectrometry analysis after ion exchange and size exclusion chromatography identified multiple proteases as possible candidates and aspartyl aminopeptidase, DNPEP, shared all the properties of the kidney lysate activity. Furthermore, recombinant GST‐DNPEP produced similar proteolytic pattern. No mouse knockout model was, however, available to be used as negative control. In this study, we used a DNPEP‐mutant mouse generated by EUCOMM as well as a novel CRISPR/cas9 mouse knockout to assess the activity of their kidney lysates towards SPAK. Two mouse models had to be used because different anti‐DNPEP antibodies provided conflicting data on whether the EUCOMM mouse resulted in a true knockout. We show that in the absence of DNPEP, the kidney lysates retain their ability to cleave SPAK, indicating that DNPEP might have been misidentified as the protease behind the kidney lysate activity, or that the aspartyl aminopeptidase might not be the only protease cleaving SPAK in kidney.

## Introduction

A sizable (30–40%) fraction of the Na^+^ delivered to the urine during glomeruli‐mediated plasma filtration is jointly reclaimed in the thick ascending limb of Henle (TALH) and the distal convoluted tubule (DCT), and this reabsorption is tightly regulated (Ares et al. 2011; Subramanya and Ellison 2014). The mechanisms of Na^+^ reabsorption are the basolateral Na^+^/K^+^ pump, which provides the transport driving force, and the apical bumetanide‐sensitive Na‐K‐2Cl cotransporter‐2, NKCC2 in the TAL, and the thiazide‐sensitive Na‐Cl cotransporter, NCC in the DCT (Gamba 2005a). The apical transporters limit the rate of Na^+^ and Cl^−^ reabsorption. They are activated by signaling cascades that involves the with‐no‐lysine (WNK) kinases WNK1 and WNK4 and the Ste20p‐like kinases SPAK and OSR1 (Gamba 2005a; Gimenez and Forbush 2005; Welling et al. 2010).

Disruption of NKCC2 and NCC function in their respective segments lead to significant changes in the amount of salt that is reabsorbed with possible effects on blood pressure, but also to indirect changes in plasma K^+^, Ca^2+^, and Mg^2+^ concentrations as well as blood pH. Indeed, loss‐of‐function mutations in human NKCC2 and NCC cause Bartter and Gitelman syndromes, respectively, which are salt wasting disorders characterized by hypokalemia and blood pH disturbances (Simon et al. 1996a,b). In contrast, NCC gain‐of‐function due to mutations in WNK kinases (Wilson et al. 2001) or in the E3 ubiquitin ligase Cullin 3 or its adaptor protein Kelch‐like‐3 (Boyden et al. 2012) cause Gordon syndrome, also known as Familial Hyperkalemic Hypertension (Fhht), or pseudohypoaldosteronism type II. This disorder features an opposite phenotype to loss of NCC function, that is reduced salt excretion, high blood pressure, hyperkalemia, metabolic acidosis, and hypercalcuria.

Mouse models also have contributed to our understanding of the signaling pathway regulating NKCC2 and NCC function. While OSR1 knockout mice recapitulate Bartter's phenotype (Takahashi et al. 2000), SPAK knockout and inactive SPAK knock‐in mice recapitulate Gitelman's phenotype (Rafiqi et al. 2010; Yang et al. 2010a; McCormick et al. 2011; Grimm et al. 2012). In contrast, constitutively active SPAK mice show characteristics of Gordon's syndrome (Grimm et al. 2017). Similarly, WNK4 knockout mice exhibited a mild Gitelman‐like phenotype (Castañeda‐Bueno et al. 2012), whereas a Gordon syndrome‐like phenotype is observed in mice with WNK4 gain‐of‐function (Lalioti et al. 2006; Yang et al. 2010a; Chiga et al. 2011; Chowdhury et al. 2013).

While the role of SPAK in the DCT is relatively well understood, the role of the ancestor kinase OSR1 in this segment is far less understood. The two Ste20p‐like kinases are highly similar with 96% conserved kinase domains and 67% conserved carboxyl‐termini. One region that is far less conserved is the region upstream of the catalytic domain, which is short in OSR1 and longer in SPAK which contains a proline‐/alanine‐rich region (Gagnon and Delpire 2012). Western blot analyses of kidney lysates have shown that SPAK, but not OSR1 exists not only as a full‐length kinase, but also as smaller fragments mainly located in the medulla (McCormick et al. 2011; Grimm et al. 2012). The function of these fragments is unknown, but it has been speculated that they might serve as endogenous inhibitors to the full‐length kinases, in this case mostly full‐length OSR1 (Yang et al. 2010b; McCormick et al. 2011). The origin of the fragments is also controversial. In one study Park and coworkers identified a transcript that yields a short kidney‐specific SPAK fragment (Park et al. 2013). Recently, we proposed that posttranslational proteolytic cleavage also produces SPAK fragments (Markadieu et al. 2014). Using ion exchange and size exclusion chromatography followed by MS/MS analysis, we identified the aspartyl‐aminopeptidase, DNPEP, as top candidate for the kidney‐enriched proteolytic activity. While recombinant GST‐DNPEP fusion protein resulted in SPAK cleavage, we did not possess at the time a knockout animal to serve as negative control.

In this study, we utilized two mouse models to address the specificity of the DNPEP effect. Kidney lysates were isolated from DNPEP‐mutant mice generated by EUCOMM and from DNPEP knockout mice generated by CRISPR/Cas9‐mediated gene editing. The two mouse lines were needed because of contradictory data obtained with two separate DNPEP antibodies. Data show that proteolytic digest still occurs in the absence of DNPEP expression, indicating that DNPEP is not the only protease that cleaves SPAK in kidney.

## Material and Methods

### Mice

All mouse procedures were approved by the Vanderbilt Animal Care and Use Committee. Mice were maintained in a pathogen‐free barrier facility in ventilated cages with free access to water and food. Per IACUC policy, a maximum of five mice were maintained per cage. Standard care was provided by the Division of Animal Care and laboratory personnel. Breeding was done using trios (one male plus two females) per cage and males were removed from the breeding cages on day 18 prior to the earliest possible time of delivery. Huts and nesting material were provided as enrichment. Both males and females were used in experiments.

### Mouse kidney lysates

Kidneys from wild‐type of mutant mice were removed, weighted, and promptly homogenized on ice in 500 *μ*L/150 mg tissue of lysis buffer containing 125 mmol/L NaCl, 10% glycerol, 1 mmol/L EGTA, 1 mmol/L EDTA, 1 mmol/L PMSF, 10 *μ*mol/L leupeptin, 4 *μ*mol/L aprotinin, 10 *μ*mol/L pepstatin, 1% Triton X‐100, 0.5% SDS, 20 mmol/L HEPES, pH 7.4 using a borosilicate glass with Teflon pestle. Following mechanical homogenization, samples were rotated at 4°C for 20 min and then centrifuged at 13,000*g* for 20 min at 4°C. Soluble lysates were then collected and pellets discarded.

### Proteolytic digestions

Reactions (40 *μ*L total) consisted of 2 *μ*L of kidney lysate (35 *μ*g/*μ*L), 2 *μ*L of SPAK fusion protein (5 *μ*g/*μ*L) and 36 *μ*L HEPES buffer (50 mmol/L Hepes, 140 mmol/L NaCl, pH 7.4). They were incubated at 37°C for 1 h prior to addition of sample buffer and denaturation.

### SDS‐PAGE and western blot analysis

Protein samples were denatured in 2× loading buffer at 75°C for 15 min and separated on 10% SDS‐polyacrylamide gel. The proteins were then electroblotted onto 0.45 *μ*m polyvinylidene fluoride membranes (ThermoFisher Scientific) using a semidry transfer process, and membranes were incubated for 2 h at room temperature in blocking solution containing 5% nonfat milk in TBST (150 mmol/L NaCl, 10 mmol/L TrisCl, 0.5% Tween 20). Incubation of primary antibody (1:1000) was performed overnight at 4°C. Antibodies were antiaspartyl aminopeptidase (Abcam rabbit monoclonal antibody clone EPR10301 cat#: ab154805, or Abgent mouse monoclonal antibody clone 2F9‐3A7) and rabbit anti‐mouse C‐terminal SPAK polyclonal antibody (Piechotta et al. 2002). Membranes were thoroughly washed in TBST for 3–4 h, then incubated with horseradish peroxidase‐conjugated secondary antibodies in blocking solution (1:5000) for 1 h at room temperature, and washed again thoroughly for 2 h in TBST. Protein bands were visualized by chemiluminescence (ECL Plus, Amersham Biosciences).

### CRISPR/Cas9‐mediated DNPEP knockout mice

A 20 bp sequence (AGCTCCTGAAGTTCGTGAAC, boxed in Fig. 3A) and followed by CGG as proto‐spacer adjacent (PAM) motif was selected to guide Cas9 to break DNA within exon 2 of mouse *Dnpep*. Complementary oligonucleotides flanked by partial *Bbs*I sites were annealed and ligated at *Bbs*I sites in pX330 to create target‐specific guide RNA. This vector expresses the guide RNA under a strong U6 promoter and cas9 under a hybrid chicken beta‐actin (Cbh) promoter. The modified pX330 vector was injected alongside a 191 bp sense repair oligonucleotide into 590 mouse embryos. The repair oligo contained 89 bp homology arms, a T > A mutation substituting Lys21 into a stop codon, the introduction of a *Bsp*HI restriction site and a few additional base mutations to prevent targeting of cas9 to the repair DNA (Fig. 3C). Of 590 embryos injected, 396 were transferred to 15 pseudopregnant females generating 35 pups.

### Genotyping of CRISPR/cas9 mice

Mice generated by injection of CRISPR reagents in 0.5‐day‐old embryos were genotyped by PCR at weaning age. Briefly, genomic DNA from 2 to 5 mm tail tip was extracted by incubated the tissue in 200 *μ*L 25 mmol/L NaOH + 0.2 mmol/L EDTA solution at 95°C for 45 minutes. After cooling the samples down, 200 *μ*L of 40 mmol/L Tris‐HCl (pH 5.0) was added to neutralize the base and the samples were microcentrifuged for 6 min at 16,000 *g*. PCR reactions of 25 *μ*L or 60 *μ*L were run, depending upon the genotyping protocol. To assess genotype by digest, 25 *μ*L reactions were prepared with 18.8 *μ*L water, 2.5 *μ*L buffer, 0.5 *μ*L dNTPs (10 mmol/L each), 0.5 *μ*L forward primer: GACAAAA CTGGGTGGAGGTCCTC, 0.5 *μ*L reverse primer: TTGTTTTCTGGTACGATATCCCAG, 0.2 *μ*L Taq DNA polymerase and 2 *μ*L tail DNA. Annealing temperature was set at 60°C and number of cycle set at 35. Following the PCR, 10  *μ*L of the PCR reaction was digested with 0.25 *μ*L *Bsp*HI in 30 *μ*L total volume for 2 h at 37°C. Samples were then run by 1.5% agarose gel electrophoresis. To assess genotype by sequencing, 60 *μ*L reactions were prepared with each reagent proportional to the smaller volume PCR. After PCR, 10 *μ*L of the reaction was subjected to agarose gel electrophoresis to confirm the presence of the 420 bp fragment, and the remaining 50 *μ*L was cleaned using Qiaquick PCR purification kit minicolumn (Qiagen), the DNA quantitated by measuring optical densities at 260, 280, and 320 nm, and the fragment was sequenced using the forward primer.

## Results

**Figure 1:**
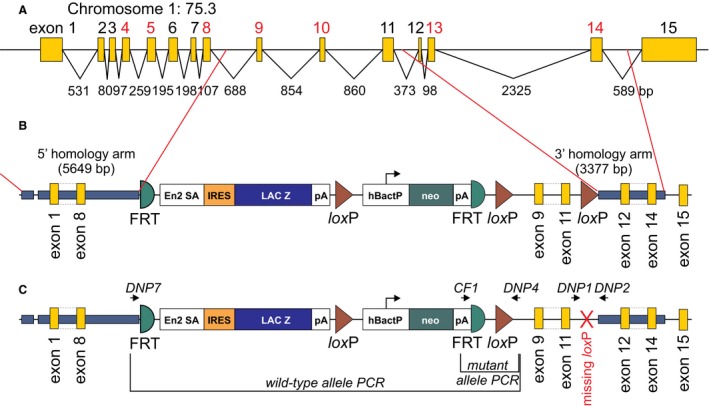
Design of the EUCOMM Dnpep mouse. (A) The Dnpep gene consists of 15 exons spaced within a relatively small 10 kb genomic stretch of mouse chromosome 1. Many exons (numbered in red font) are multiple of 3 or cassette exons. Their removal does not affect the open reading frame of the protein. (B) The construct consisted of 5.6 kb of 5′ arm of recombination, *β*‐galactosidase and neomycin‐resistance gene cassettes flanked by *Frt* recombination sites, followed by two *lox*P sites surrounding exons 9–11, and a smaller 3.4 kb 3′ arm of recombination. (C) Structure of the mutant allele in the Dnpep‐targeted mouse. Note the missing loxP site, confirmed by sequencing using primers DNP1 and DNP2. The location of the genotyping PCR primers DNP7, DNP4, and CF1 is indicated by arrows. Frt: Flippase recognition target; En2‐SA: splice acceptor from En‐2 gene; IRES: Internal Ribosomal Entry Site; LAC Z: *β*‐galactosidase open reading frame; pA: poly adenylation signal; loxP: bacteriophage P1 original sequence recognized by CRE recombinase; hBactIP: autonomous promoter; neo: neomycin resistance open reading frame.

To address the specificity of the SPAK fragments to DNPEP, we purchased from the Sanger center an available Dnpep targeted mouse line (Dnpep^tm1e(EUCOMM)Wtsi^). To create the mouse, embryonic stem cells were targeted by homologous recombination with a construct designed to insert loxP sites around exons 9–11 (Fig. 1A–B). Unfortunately, the ES cell clone *EPD0821_6_H02* used to produce the mouse failed to integrate the most 3′ loxP site. This was likely due to the 2.7 kb region of exons 9–11 serving as the small arm of recombination instead of the Dnpep gene fragment located downstream of the last loxP site in the construct. Thus, while the lacZ and neomycin cassettes could readily be removed using FlpE‐mediated recombination (Fig. 1C), the mouse produced would have contained a unique loxP site upstream of exon 9 and would have thus failed to produce a knockout mouse upon CRE‐mediated recombination.

**Figure 2:**
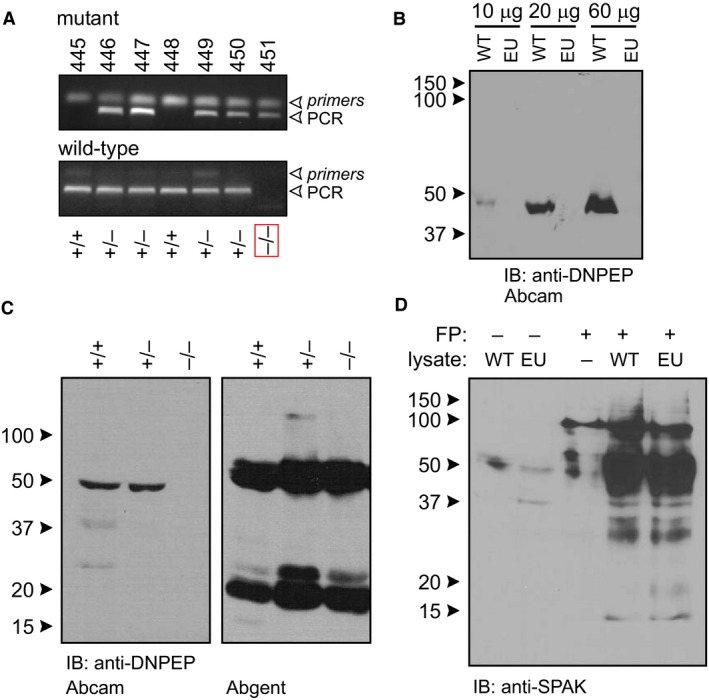
DNPEP expression and cleavage activity in Dnpep homozygous mice. (A) PCR genotyping of 7 pups of Dnpep heterozygous cross. Top picture represents the mutant allele (CF1‐DNP4 primers) and bottom picture represents the wild‐type allele (DNP7‐DNP4 primers). Sizes of PCR fragments are 226 bp (mutant) and 324 bp (wild‐type), respectively. All three anticipated genotypes are observed. Homozygote mutant are highlighted in red box. (B) Western blot analysis with increasing amount of kidney protein lysate from wild‐type and EUCOMM (EU) mutant mouse using an anti‐DNPEP antibody from Abcam. (C) Comparison between Abcam and Abgent antibodies on protein samples isolated from wild‐type (+/+), heterozygote EUCOMM (±), and EUCOMM homozygote (‐/‐) mouse kidneys. *D*. GST‐SPAK (100 kDa) fusion protein (FP) is cleaved into smaller bands with both wild‐type (lane 4) and Dnpep EUCOMM mutant (lane 5) kidney lysates. The intense ~50 kDa band does not come from kidney lysate as identical lysate samples are loaded in lanes 1 and 2.

Next, we reasoned that the insertion of a large 7 kb DNA fragment between exons 8 and 9 of Dnpep in this mouse might be sufficient to disrupt normal splicing and production of the DNPEP protein. If true, the knockout could be obtained by crossing directly two Dnpep^tm1e(EUCOMM)Wtsi^ mice. The first litter of seven pups produced four heterozygotes, two wild‐type, and one homozygote mice (Fig. 2A). Western blot analysis using an antibody purchased from Abcam showed a band at a molecular weight slightly lower than 50 kDa in wild‐type kidney but not in knockout kidney (Fig. 2B). Because the predicted molecular size is 52.5, we utilized a second antibody purchased from Abgent. This antibody recognized a band slightly larger, but present in kidneys from control, heterozygous, and homozygous mice (Fig. 2C). In addition, there were 1–2 bands observed at lower molecular sizes. This experiment provides conflicting data as to whether the EUCOMM mouse is a true knockout and indicates that the Abgent antibody might be unspecific.

To assess proteolytic cleavage on SPAK, lysates were isolated from wild‐type and homozygote‐mutant kidneys and incubated in the presence or absence of a recombinant GST‐SPAK fusion protein (Markadieu et al. 2014). Following the reaction, proteins were separated by SDS‐PAGE, transferred to membrane and subjected to Western blot analysis using a SPAK‐specific antibody (Piechotta et al. 2002). As seen in Figure 2D, the GST‐SPAK fusion protein runs close to 100 kDa (lane 3) and is cleaved similarly by protein lysates isolated from wild‐type or homozygous DNPEP‐mutant kidneys.

**Figure 3:**
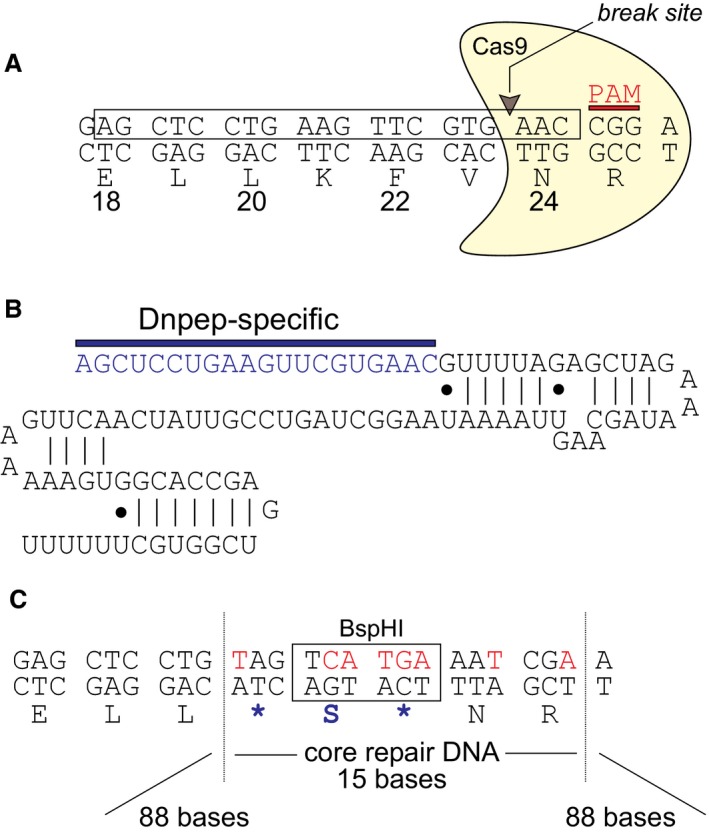
Design of a CRISPR/Cas9 *Dnpep* knockout mouse. (A) Design of the guide RNA around amino acids 18–24, which are encoded by exon 2. The PAM sequence consists of the Arg25 codon (CGG). Cas9 and the break site are indicated. (B) Sequence of the guide RNA with DNPEP‐specific target sequence followed by trace RNA. (C) Several mutations indicated in red font in the upper strand are created in the core repair DNA to introduce a stop codon (Lys21Ter) and a *Bsp*HI restriction site, and prevent Cas9 to recognize the repair DNA. The repair DNA consists of a 191 base single‐stranded oligonucleotide with a 15 bp core repair sequence flanked by 88 base arms of recombination.

**Figure 4:**
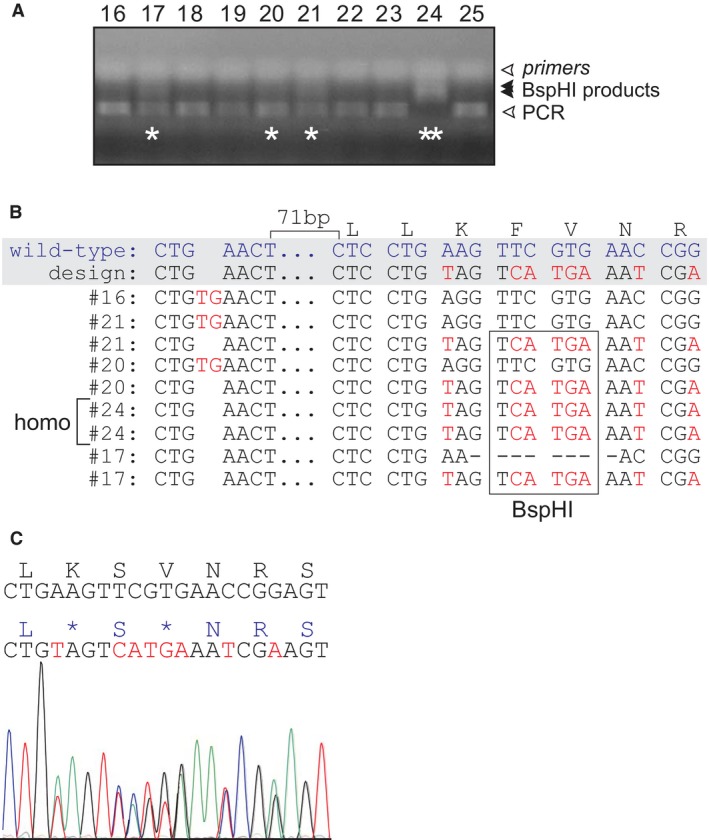
Generation of the CRISPR/Cas9 *Dnpep* knockout mouse (A) Agarose gel showing genotyping of 10 CRISPR/cas9 offsping. An aliquot of the PCR reaction was digested with *Bsp*HI. Note the full‐length PCR fragment (lower band), digested products in samples labeled with a star, and absence of full‐length product in sample #24, indicating homozygosity. (B) Sequence of the two alleles of five mice showing mutations in red. The designed allele with stop codon (TAG) and *Bsp*HI restriction site is found in four of these mice. (C) Chromatogram of one heterozygous offspring showing germline transmission with one wild‐type sequence and one mutant sequence.

Because we were unsure of the specificity of the antibodies and the knockout nature of the mouse model, we needed an independent model to validate the data obtained with the DNPEP EUCOMM mouse. We thus created our own knockout mouse. Using CRISPR/Cas9 technology, we targeted exon 2 of Dnpep to introduce a premature stop codon and thus disrupt translation of a full‐length peptidase (Fig. 3A–C). Embryo injections resulted in the production of 31 live offspring, which were genotyped at weaning by amplifying a 420 bp fragment from tail genomic DNA, followed by *Bsp*HI digest (Fig. 4A). Seven *Bsp*HI‐sensitive (positive) animals out of 31 (23%) were identified and PCR fragments were amplified again, purified, and sequenced (Fig. 4B). Out of the 7 mice, two mice displayed multiple peaks in the chromatograms that indicated chimerism. Three mice (#17, 20, 21 in Fig. 4B) were compound heterozygotes with one allele carrying the designed mutation; one mouse (#16) had a wild‐type allele and the allele carrying a 2 bp addition some 89 bp upstream of the CRISPR cut site; and one animal (#24) was homozygous for the designed mutated allele (see Fig. 4B). We selected two lines and crossed them to C57BL/6J mice twice to demonstrate germline transmission (Fig. 4C) and eliminate possible off target events. Heterozygous mice were then crossed to generate homozygous knockouts.

**Figure 5:**
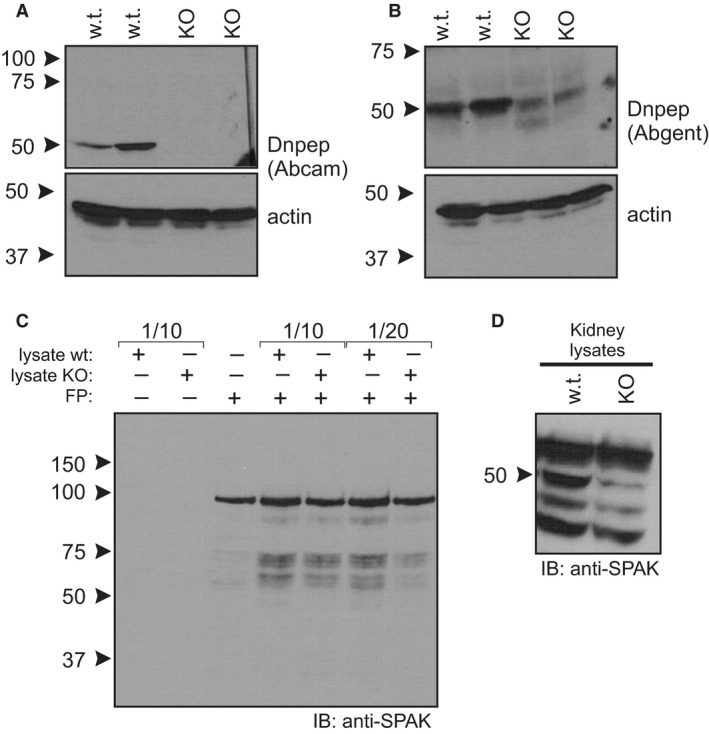
Western blot analysis of DNPEP wild‐type and knockout kidneys. (A) Presence of a band at ~50 kDa in kidneys from wild‐type mice but not Dnpep knockout mice is seen with the anti‐Dnpep Abcam antibody. Actin labeling demonstrated equal labeling. (B) Identical experiment was performed using anti‐Dnpep antibody from Abgent. While the signal was somewhat weaker in the knockout, there was still very significant signal at the ~50 kDa molecular size. Actin signal confirmed equal loading. (C). The GST‐SPAK fusion protein (100 kDa, lane 3) is cleaved into smaller bands with both wild‐type (lanes 4, 6) and Dnpep knockout (lane 5, 7) kidney lysates. (D) Western blot analysis of SPAK in kidneys isolated from wild‐type and DNPEP knockout mice. FP, Fusion Protein; w.t., samples from wild‐type mice; KO, samples from CRISPR/cas9‐generated DNPEP knockout mice.

Using this new mouse line that clearly disrupted translation at the beginning of the protein (at amino acid 25), we retested the two anti‐DNPEP monoclonal antibodies and observed data similar to those obtained with the samples isolated from the EUCOMM homozygous mouse (Fig. 5A and B). Similarly, lysates from our homozygous DNPEP knockout mice retained cleavage activity of GST‐SPAK fusion protein (Fig. 5C), indicating that a protease other than DNPEP also mediates proteolytic cleavage of SPAK. Top confirm presence of SPAK fragments in native tissue, we performed a Western blot using lysates isolated from wild‐type and DNPEP knockout mice. As seen in Figure 5D, similar pattern with full‐length kinase and fragments is observed in DNPEP knockout and wild‐type kidney samples.

## Discussion

We recently reported that the renal aspartyl aminopeptidase DNPEP exerts proteolytic cleavage activity against SPAK but not OSR1 (Markadieu et al. 2014). The sites of cleavage were located within the proline/alanine‐rich region but also within the kinase domain. Evidence of DNPEP's involvement included its presence in ion exchange and size exclusion chromatography fraction that demonstrated cleavage activity, as well as its pharmacological profile. In addition, SPAK proteolytic cleavage activity required the presence of Zn^2+^ as a cofactor, strengthening the case for DNPEP as it is known to be a Zn^2+^ metalloprotease. Finally, recombinant GST‐DNPEP fusion protein isolated from bacteria demonstrated cleavage activity, whereas bacteria lysates containing GST alone did not. Although the evidence was overwhelming, our study did not include kidney lysate isolated from DNPEP knockout mice, as these mice were not available.

Since the publication of our original paper, we obtained a mouse that was made available by the European Conditional Mouse Mutagenesis (EUCOMM) Program. Unfortunately, recombination in embryonic stem cells was not complete and the third loxP site was not inserted (Fig. 1B and C). Failure of a loxP to insert is a well‐documented weakness of recombination, especially when the region between two loxP sites is large enough to be used by the cell as arm or recombination. While the vendor (The Wellcome Trust Sanger Institute) was fully transparent about the lack of conditionality of this mouse model, we still obtained the mouse hoping that the insertion of a large foreign DNA fragment within intron 8 would disrupt splicing and gene transcription, resulting in the absence of a protein in the homozygous state.

While breeding the mice to homozygosity was relatively easy, demonstrating DNPEP knockout was far more challenging due to conflicting results between two anti‐DNPEP antibodies. While the Abcam antibody (directed against portions of exon 12 and 13 per manufacturer) demonstrated absence of protein at ~50 kDa in the homozygote mouse, the Abgent antibody (with unknown epitope, but the entire protein was used to generate the antibody) demonstrated bands at also ~50 kDa, but in all genotypes. While the data suggested that the Abcam antibody might be specific to DNPEP, whereas the Abgent antibody not, because all the exons were still present in the homozygous mouse, no definitive conclusion could be drawn from these data.

The project remained uncertain until CRISPR/Cas9 allowed us to generate mouse models within a few months. We were indeed successful in creating within 3–4 months a knock‐in mutation that mimics a human mutation in *SLC12A6*, the gene encoding the K‐Cl cotransporter‐3 (Kahle et al. 2016). To resolve the uncertain EUCOMM knockout mouse and contradictory antibody data, we decided to create our own DNPEP knockout mouse using CRISPR/cas9. As indicated in Figure 4, we were successful in substituting the codon encoding lysine residue 23 (for DNPEP isoform 475 aa) into a stop codon, leading to a DNPEP‐K23ter‐mutant mouse. Early open reading frame termination guaranties that no functional protein is being expressed. We were then able to demonstrate viability and no overt phenotype of the homozygous mouse.

Using this mouse, we repeated the Western blot analysis with the two antibodies and were able with this independent model to confirm that the Abcam antibody is specific to DNPEP, and to demonstrate that the Abgent antibody recognizes a protein of similar size, but different identity. These latest data also confirmed that the homozygous EUCOMM mouse was indeed a knockout mouse as it was lacking the epitope (exons 12–13) which encode key domains of the peptidase and likely to disrupt the overall homododecamer tetrahedron structure of the protein.

Overall, these new data question the idea of DNPEP being the protease cleaving SPAK in kidney. It is still unclear whether multiple proteases are at play and elimination of one protease has minimal effect on the ability of kidney lysate to produce the SPAK fragments, or we misidentified the protease. It is important to stress that if DNPEP is not the protease, the one at play must have characteristics that are very similar to it, that is, very high‐molecular‐weight protein, coeluting in the same range of salt concentrations, sensitivity to Zn^2+^, inhibition by phenanthroline, DTT, and high concentrations of EDTA. Note that the mass‐spectrometry data in our original publication identified additional proteases in the fractions containing the proteolytic activity. The list included among others: Lap3 (or cytosol aminopeptidase), cathepsin B, meprin A (Markadieu et al. 2014). While Lap3 was the top candidate based on mass‐spectrometry fragments abundance, its high sensitivity to bestatin disqualified it as a candidate.

## Conflict of Interest

None declared.
